# Efficacy of Immune Checkpoint Blockade in Advanced Upper Tract Urothelial Cancer With DNA Mismatch Repair Deficiency or Microsatellite Instability

**DOI:** 10.1200/PO-25-00772

**Published:** 2026-03-30

**Authors:** Mohammad Jad Moussa, Alexander Y. Andreev-Drakhlin, Aradhana M. Venkatesan, Surena F. Matin, Lianchun Xiao, Rebecca S.S. Tidwell, Amishi Y. Shah, Ana C. Adriazola, Leah Shaw, Jianjun Gao, John K. Lin, Sangeeta Goswami, Pavlos Msaouel, Charles C. Guo, Nizar M. Tannir, Arlene O. Siefker-Radtke, Omar Alhalabi, Matthew T. Campbell

**Affiliations:** ^1^ Department of Genitourinary Medical Oncology, The University of Texas MD Anderson Cancer Center, Houston, TX; ^2^ Internal Medicine Residency Program, Department of Medicine, Baylor College of Medicine, Houston, TX; ^3^ Genentech, South San Francisco, CA; ^4^ Department of Radiology, The University of Texas MD Anderson Cancer Center, Houston, TX; ^5^ Department of Urology, The University of Texas MD Anderson Cancer Center, Houston, TX; ^6^ Department of Biostatistics, The University of Texas MD Anderson Cancer Center, Houston, TX; ^7^ Department of Genitourinary Radiation Oncology, The University of Texas MD Anderson Cancer Center, Houston, TX; ^8^ Department of Pathology, The University of Texas MD Anderson Cancer Center, Houston, TX

## Abstract

**PURPOSE:**

Deficient DNA mismatch repair (dMMR) and microsatellite instability-high (MSI-H) status, which sensitizes tumors to immune checkpoint inhibitors (ICIs), is three times more common with upper tract urothelial carcinoma (UTUC) than with bladder cancer. However, data on ICI efficacy against dMMR/MSI-H advanced UTUC remain limited.

**MATERIALS AND METHODS:**

We retrospectively reviewed records of 24 patients with dMMR/MSI-H advanced UTUC treated with single-agent ICIs at a single institution (2015-2024). Descriptive statistics and the Kaplan-Meier method for survival outcomes were used.

**RESULTS:**

Immunohistochemistry confirmed dMMR in 22 (92%) patients, with loss of MSH2 or MSH6 in 15 (68.2%) patients and loss of PMS2 or MLH1 in seven patients (31.8%). Germline mutation testing confirmed Lynch syndrome in 16 (67%) patients. ICI monotherapy was associated with a median progression-free survival (PFS) time of 65.9 months (95% CI, 31.6 months to nonevaluable [NE]). The PFS rates at 12 and 24 months were 95.2% (95% CI, 86.1% to 100.0%) and 78.8% (95% CI, 60.1% to 97.5%), respectively. At a median follow-up duration of 56.9 months (95% CI, 42.2 to 92.2 months), the median overall survival time was not reached (95% CI, 65.9 months to NE). The confirmed overall response rate was 83%, including 16 complete responses. Four (17%) patients were offered surgical consolidation with these pathologic outcomes: ypTaN0, ypT0N0, and ypT1N0 (two patients). Eight patients (33%) experienced grade ≥3 immune-related adverse events, including bullous pemphigoid (n = 3), hepatitis (n = 1), pancytopenia (n = 1), colitis (n = 1), polyendocrinopathy (n = 1), and polyarthritis with sarcoid-like reaction (n = 1).

**CONCLUSION:**

Our hypothesis-generating findings suggest that dMMR/MSI-H may serve as a biomarker of sensitivity to single-agent ICIs in advanced UTUC. External validation in larger, ideally prospective, studies is needed to confirm the effectiveness and durability of immune checkpoint blockade in this molecular subgroup.

## INTRODUCTION

Deficient DNA mismatch repair (dMMR) arises from loss of one or more MMR proteins (MLH1, MSH2, MSH6, and PMS2),^1^ leading to a high frequency of nucleotide gain or loss in microsatellite regions, known as microsatellite instability (MSI). This immunogenic phenotype enhances immune recognition and increases the responsiveness of dMMR/MSI cancers to treatment with immune checkpoint inhibitors (ICIs) like pembrolizumab and dostarlimab, which received tissue-agnostic drug approval for treatment of multiple tumor types.^2,3^ Whereas pembrolizumab was initially approved for treatment of platinum-ineligible metastatic urothelial carcinoma (UC),^4^ current treatment with it often involves combination therapy with enfortumab vedotin (EV), albeit at a high financial burden and with a new spectrum of toxicity.^5^ Although the current treatment paradigm for locally advanced (LA) or metastatic upper tract UC (UTUC) mirrors that of advanced UC, the predictive and prognostic impacts of dMMR/MSI-high (MSI-H) status on ICI responses of LA or metastatic UTUC remain poorly documented. Notably, cases of dMMR/MSI-H status are three times more common with UTUC (dMMR, 8.95%; MSI-H, 8.36%) than with bladder cancer (dMMR, 3.09%; MSI-H, 2.11%), with the majority associated with Lynch syndrome.^6,7^ Herein we report our experience with single-agent ICIs in patients with dMMR/MSI-H advanced UTUC, in which we hypothesized that these patients would have exceptional response rates and durable remissions.

**CONTEXT**

**Key Objective**
Deficient DNA mismatch repair (dMMR)/microsatellite instability-high (MSI-H) status is enriched in upper tract urothelial carcinoma (UTUC) compared with bladder cancer and represents an actionable biomarker for immune checkpoint inhibitor (ICI) therapy.
**Knowledge Generated**
In this first and largest single-institution series of patients with dMMR/MSI-H advanced UTUC to date, single-agent ICIs achieved high complete response rates, durable remissions, and even treatment-free intervals. The durable responses observed in this cohort, which was enriched for patients with Lynch syndrome, highlight the utility of routine molecular testing to identify dMMR/MSI-H UTUC early in the disease course. Our findings support integrating biomarker-driven ICI therapy into standard management in the genitourinary oncologist's clinic and exploring its role in organ-sparing strategies, like kidney preservation.
**Relevance**
Our work bridges biomarker investigation with therapeutic application in a rare cancer setting where clinical trials are challenging, providing a foundation to refine sequencing, treatment duration, and toxicity management of ICI therapy in dMMR/MSI-H UTUC.

## MATERIALS AND METHODS

Data for an institutional cohort of 119 consecutive patients with advanced UTUC treated at The University of Texas MD Anderson Cancer Center from January 2015 to December 2024 were collected and analyzed. LA or unresectable UTUC was defined as nonmetastatic (M0) disease with high T or N stage (eg, T3-T4 and/or N1-N2) deemed unsuitable for curative surgery by a multidisciplinary team due to tumor extent, local invasion, or patient-related factors. Metastatic UTUC was defined as M1 disease per AJCC 8th edition TNM staging, indicating distant metastases to nonregional lymph nodes or distant organs (lungs, liver, or bone). The study was approved by the MD Anderson Institutional Review Board (protocol no. PA16-0736).

The inclusion criterion was dMMR/MSI-H status confirmed using (1) polymerase chain reaction (PCR)–based detection of MSI using five microsatellite markers, including at least BAT-25 and BAT-26, or (2) immunohistochemistry (IHC) performed with formalin-fixed, paraffin-embedded tumor sections using antibodies against MLH1, MSH2, MSH6, and PMS2. To identify cases associated with Lynch syndrome, germline testing for mutations in MMR genes and *EPCAM* was performed using an Invitae Lynch Syndrome Panel (San Francisco, California, USA) or an Ambry Genetics Lynch Syndrome Panel (Aliso Viejo, California, USA). Whenever available, complementary next-generation sequencing results for tumor-associated somatic mutation profiling of the MMR genes or *POLE* mutations were reported.

The primary end points included the objective response rate (ORR) determined using RECIST v1.1, overall survival (OS), progression-free survival (PFS), and the 12- and 24-month PFS rates. The methods of estimating these parameters and other time-to-event outcomes are detailed in Appendix (Table A1).

### Ethics Approval and Consent to Participate

The study was performed in accordance with protocol PA16-0736, approved by the MD Anderson Cancer Center Institutional Review Board. The protocol is a low-risk investigational study with a waiver of consent.

## RESULTS

### Baseline Characteristics of Patients

Twenty-four patients met our inclusion criterion. Of these patients, nine (38%) had LA or unresectable UTUC, whereas 15 (62%) had metastatic disease at the start of treatment with ICIs (Table 1). We histologically confirmed pure UC in all but three specimens of mixed variant histology UC. Visceral disease was present in six (25%) patients, and most patients (20 [83%]) had a Bellmunt risk score of 0 or 1. Furthermore, most patients (83%) had a good Eastern Cooperative Oncology Group performance status (0 or 1) at the start of ICI administration.

**TABLE 1.** Baseline Characteristics of the Study Cohort (N = 24)

Variable	No. (%)
Median age at diagnosis of advanced disease, year (IQR)	66.0 (55.8-72.3)
Male sex	14 (58)
Tumor origin	
Ureter	9 (38)
Renal pelvis	15 (63)
Race/ethnicity	
White, non-Hispanic	19 (79)
Black	1 (4)
Hispanic	2 (8)
Other	2 (8)
UC mixed with variant histology	3 (13)^a^
ICI	
Pembrolizumab	17 (71)
Nivolumab	4 (17)
Atezolizumab	3 (13)
Visceral metastasis at ICI start	
Liver	1 (4)
Lung	2 (8)
Bone	4 (17)
Other	2 (8)
Any	6 (25)
ECOG PS at ICI start	
0	6 (25)
1	14 (58)
2	2 (8)
≥3	2 (8)
Hemoglobin <10 g/dL at ICI start	4 (17)
Bellmunt risk score	
0-1	20 (83)
2	3 (13)
3	1 (4)
Prior surgery for localized disease	
Nephroureterectomy^b^	4 (17)
Ureterectomy	2 (8)
No prior surgery	18 (75)
Reason to avoid surgical resection	
LA or metastatic disease	14 (58)
PS and comorbidities	1 (4)
Solitary kidney^c^	2 (8)
Provider's or patient's preference	1 (4)
Total	18 (75)^a^
Prior platinum-based chemotherapy	
Cisplatin-based	7 (29)
Non–cisplatin-based	6 (25)
Total	13 (54)
Reason to avoid chemotherapy	
Low ECOG PS and comorbidities	1 (4)
Low GFR	5 (21)
Low ECOG PS and comorbidities + low GFR	1 (4)
Patient or provider preference	4 (17)
Total	11 (46)
Best overall response to chemotherapy (*n* = 13)	
CR	1 (8)
PR	4 (31)
SD	5 (38)
PD	2 (15)
NE	1 (8)
History of previous cancer	
Bladder cancer	8 (33)
UTUC	3 (13)
Colon cancer	7 (29)
Other	6 (25)

Abbreviations: CR, complete response; ECOG, Eastern Cooperative Oncology Group; GFR, glomerular filtration rate; ICI, immune checkpoint inhibitor; LA, locally advanced; NE, nonevaluable; PD, progressive disease; PR, partial response; PS, performance status; SD, stable disease; UC, urothelial carcinoma; UTUC, upper tract urothelial carcinoma.

^a^ Three coexistent subtypes: squamous, micropapillary, and neuroendocrine.

^b^ Two patients had prior nephroureterectomy for contralateral localized UTUC.

^c^ Previous surgical resection of contralateral localized UTUC.

Most of the patients received pembrolizumab (17 [71%]), and half of them were treatment-naïve before receiving ICIs, defined as no previous systemic chemotherapy or receipt of the last chemotherapy dose more than 12 months before ICI use. Thirteen patients (54%) previously received systemic chemotherapy, defined with receipt of the last dose of chemotherapy within 12 months before ICI initiation. Among them, most (7/13, 54%) received cisplatin-based regimens, with a median glomerular filtration rate of 46.0 mL/min (IQR, 37.5-84.0 mL/min) at treatment initiation. The ORR for chemotherapy was 38% (five patients: one complete response [CR], four partial responses [PRs]); five patients had stable disease (SD), and three patients had progressive disease (PD) or nonevaluable (NE) disease. Most of the chemotherapy-exposed patients (85%) received ICIs upon disease progression, within 3 months of their last chemotherapy dose.

### Molecular Characterization

**FIG. 1.**
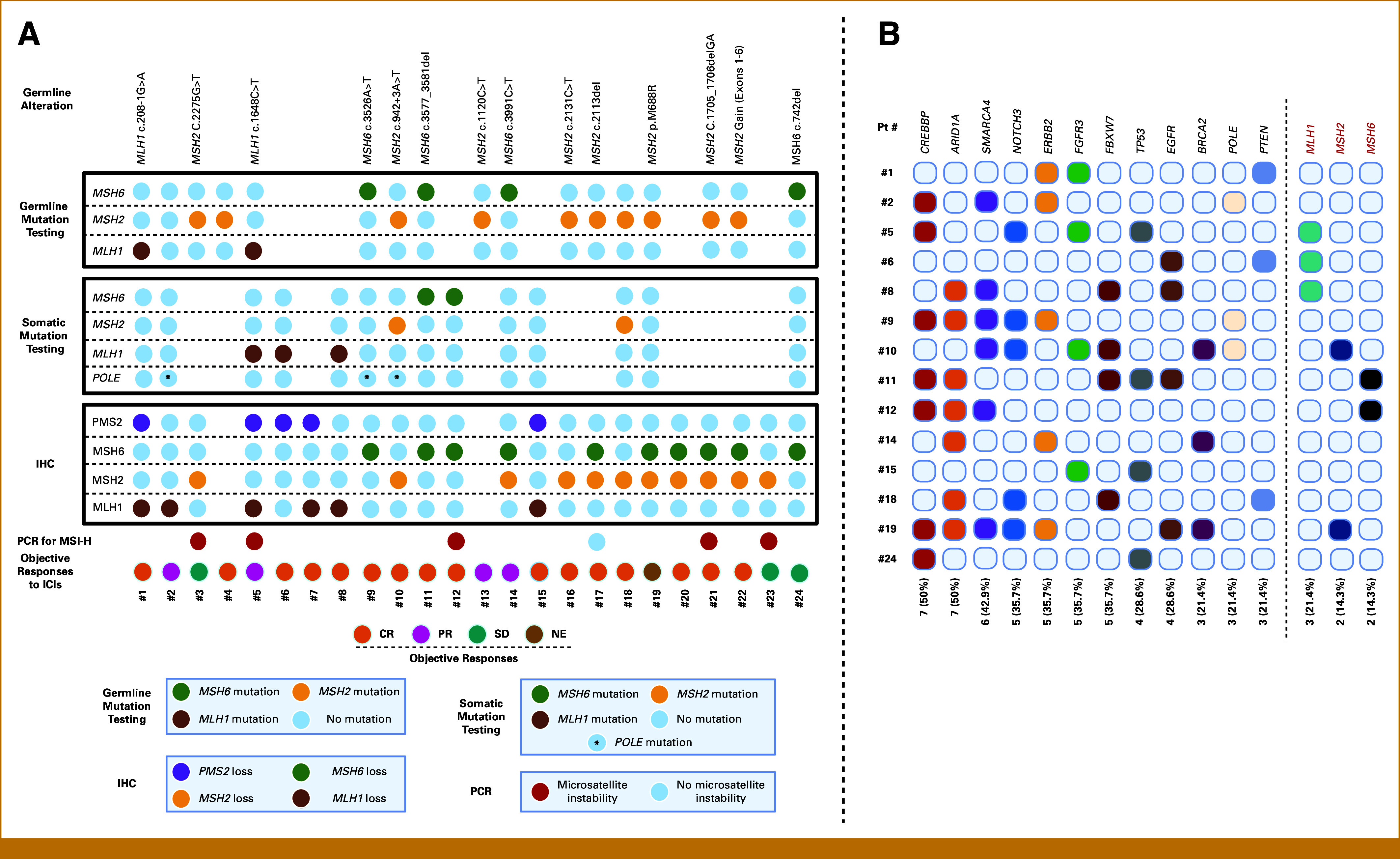
Characterization of dMMR/MSI-H status (A) and somatic alterations (B) identified using next-generation sequencing in the tumors of our cohort (N = 24). A colored circle indicates a positive test result for an alteration in the corresponding gene or protein. A gray circle indicates a negative test, whereas the absence of a circle means the test was not ordered for the patient. dMMR, DNA mismatch repair; ICIs, immune checkpoint inhibitors; IHC, immunohistochemistry; MSI-H, microsatellite instability-high; PCR, polymerase chain reaction.

We assessed the dMMR/MSI-H status of the study patients using at least one of three benchmark methodologies described earlier (Fig 1). For most patients (22 [92%]), immunohistochemical testing confirmed at least one MMR protein deficiency. Two patients were included on the basis of pathogenic germline MMR mutations despite having MMR-proficient IHC and microsatellite-stable tumors. MSH2 and MSH6 were the two most frequently lost proteins according to IHC (11/22 [50%] and 10/22 [45%], respectively). The most common patterns of paired MMR protein loss were co-loss of MSH2 and MSH6 (n = 6) and co-loss of MLH1 and PMS2 (n = 4). PCR testing was available for six patients (25%), confirming MSI-H status in five of them.

Germline mutation testing was available for 17 (71%) patients, whereas next-generation sequencing for somatic mutations was available for 14 (58%) patients. Germline testing helped diagnose Lynch syndrome in 16 (67%) patients due to mutations of *MSH2* (n = 10), *MSH6* (n = 4), and *MLH1* (n = 2). The remaining patient without germline MMR gene mutations had a somatic *POLE* mutation.

### Response and Survival Analysis

**FIG. 2.**
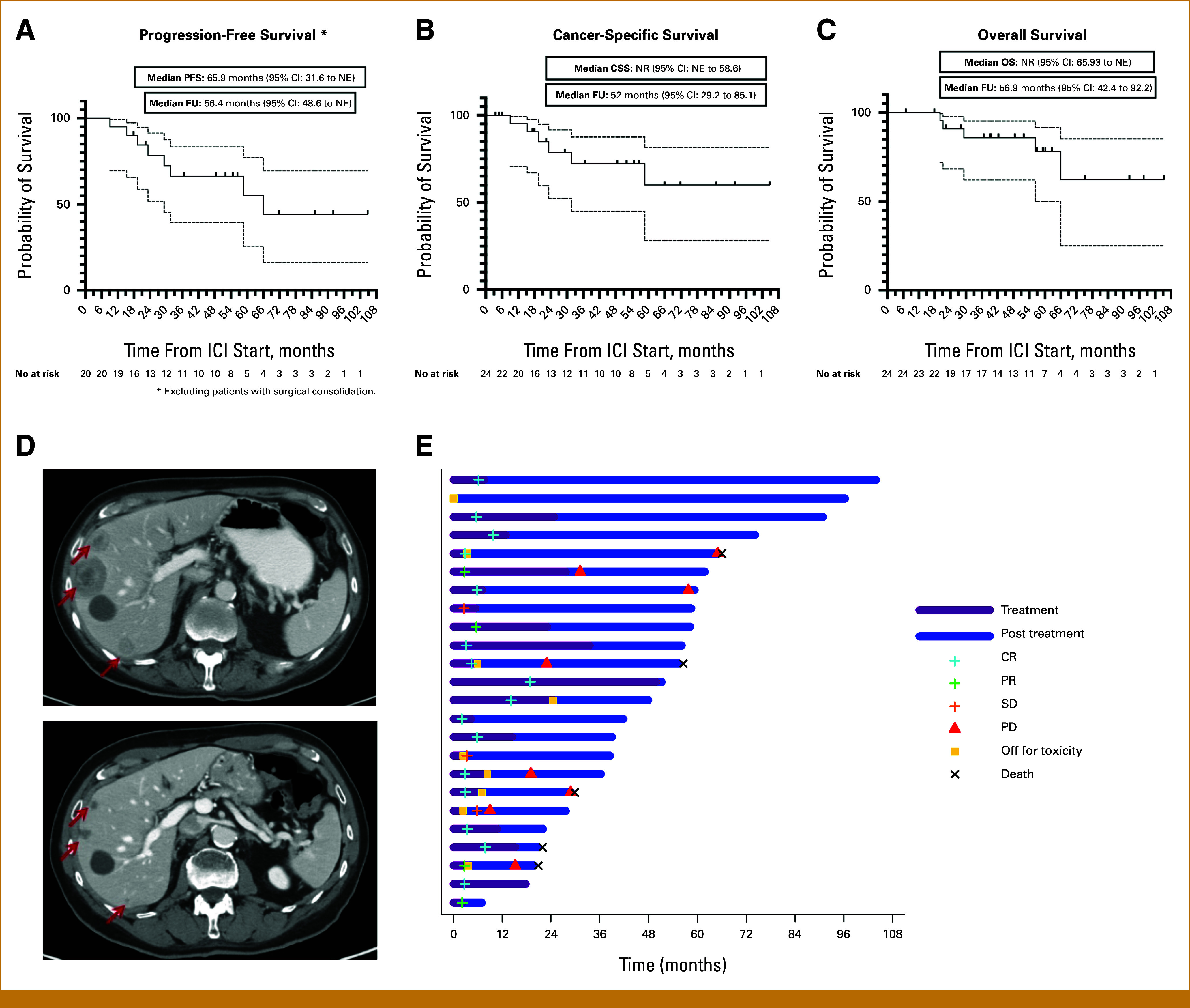
Kaplan-Meier (A) PFS, (B) CSS, and (C) OS curves for our patient cohort (N = 24). (D) Representative contrast-enhanced computerized tomography scans of the abdomen demonstrating a marked, near-complete radiologic response of liver metastases in one patient. (E) Swimmer plot of durations of treatment with ICIs and corresponding clinical outcomes. Although nine patients are depicted with toxicity events in the plot, only eight events ultimately led to treatment discontinuation. Patient 16 initially discontinued therapy for colitis; however, symptoms resolved with FMT and vedolizumab, and pembrolizumab was subsequently rechallenged successfully. Therefore, this event was not counted as a final discontinuation in the main text. CSS, cancer-specific survival; FMT, fecal microbiota transplantation; ICIs, immune checkpoint inhibitors; OS, overall survival; PFS, progression-free survival.

The median PFS duration was 65.9 months (95% CI, 31.6 months to nonevaluable [NE]) for a median follow-up time of 56.4 months (95% CI, 48.6 months to NE; Fig 2) in 20 of 24 patients (excluding four with post-ICI surgical consolidation). No patients experienced disease progression at 6 months, whereas the milestone PFS rates at 12 and 24 months were 95.2% (95% CI, 86.1% to 100.0%) and 78.8% (95% CI, 60.1% to 97.5%), respectively. Ten patients (42%) remained progression-free 4 years after ICI initiation. The median number of ICI cycles was 12.0 (IQR, 7.3-22.8), and the median time on treatment was 14.4 months (IQR, 4.7-25.0 months). The median OS time was not reached (95% CI, 65.9 months to NE), and the median follow-up time was 56.9 months (95% CI, 42.4 to 92.2 months). The median cancer-specific survival (CSS) time also was not reached (95% CI, NE to 58.6 months) for a median follow-up time of 52.0 months (95% CI, 29.2 to 85.1 months).

The confirmed ORR for the entire cohort was 83%, including 16 (67%) patients who had a radiographic CR. The median time to best response (CR/PR) was 3.9 months (IQR, 2.8-6.2 months; Table 2). Four patients underwent surgical consolidation after ICI-based therapy (three radical nephroureterectomies and one radical nephrectomy with retroperitoneal lymph node dissection [RPLND]), for which the pathologic outcomes were ypTaN0M0, ypT0N0M0, ypT1N0M0, and ypT1N0cM1-NED (no evidence of disease), respectively (Appendix, Table A2).

**TABLE 2.** Time-to-Event and Treatment Efficacy Outcomes in the Analysis of Our Study Cohort (N = 24)

Outcome	All Patients (N = 24)
ORR, No. (%)	20 (83)
CR	16 (67)
PR	4 (17)
SD	3 (13)
SD >6 months	2 (8)
PD	0
NE	1 (4)
Clinical benefit, No. (%)	22 (92)
Median OS time, month (95% CI)	Not reached (65.9 to NE)
Median follow-up time, month (95% CI)	56.9 (42.2 to 92.2)
Median PFS time, month (95% CI)	65.9 (31.6 to NE)
12-month milestone PFS rate (95% CI)	95.2% (86.1% to 100.0%)
24-month milestone PFS rate (95% CI)	78.8% (60.1% to 97.5%)
Median CSS time, month (95% CI)	Not reached (NE to 58.6)
Median duration of response, month (95% CI)	63.1 (26.3 to NE)
Median time on treatment, month (IQR)	14.4 (4.7-25.0)
Median time to best overall response, month (IQR)	3.9 (2.8-6.2)

Abbreviations: CR, complete response; CSS, cancer-specific survival; NE, nonevaluable; PD, progressive disease; PFS, progression-free survival; PR, partial response; SD, stable disease.

Eight patients (33.3%) experienced grade ≥3 immune-related adverse events (irAEs), including bullous pemphigoid (n = 3; one without concurrent organ dysfunction, one with concurrent hepatitis, and one with nephritis), hepatitis (n = 1), pancytopenia (n = 1), colitis (with grade 2 pneumonitis; n = 1), polyendocrinopathy (n = 1), and polyarthritis with sarcoid-like reaction (n = 1). The patient with colitis had a temporary discontinuation of therapy and eventually resumed treatment with pembrolizumab after treatment. A summary of irAEs is highlighted in Appendix (Table A3). Overall, treatment discontinuation was attributed to irAEs (n = 8, 33.3%; seven grade ≥3 and one grade 2), treatment completion per provider discretion (n = 10, 41.7%), surgical consolidation to optimize tumor control (n = 4, 16.7%), or disease progression (n = 1, 4.2%; Appendix, Table A4). At the time of analysis, one patient (4.2%) remained on active treatment.

Of the 24 patients, three (13%) received therapy after ICIs. Of the 21 patients who did not, 17 (71%) remained on active surveillance, one (4%) continued receiving ICI therapy, two (8%) died of noncancer causes, and one (4%) was lost to follow-up.

Patient 5 was monitored by the neurosurgery team for multifocal bone metastases with spinal cord compression; epidural disease from T2 to T4; pathologic vertebral compression fractures at T3, T7, and T10; and lumbar spine involvement. He also had liver metastases and other visceral metastatic disease at baseline. The patient received irradiation at 30 Gy in 10 fractions to T1-T12 followed by treatment with gemcitabine and cisplatin, experiencing PD as the best response. He subsequently received pembrolizumab, and a 3-month follow-up spine magnetic resonance imaging scan showed disease stability and significant response, including bone marrow reconversion and visceral improvement (Fig 2). Serial scans have shown sustained deep response. The patient remains neurologically intact, ambulatory, and under surveillance. This clinical evolution highlights a significant systemic response that mitigated critical neurologic threats in highly sensitive spinal compartments, thereby averting the need for aggressive surgical decompression.

## DISCUSSION

The results presented herein are among the first to characterize outcomes of dMMR/MSI-H LA or metastatic UTUC treated with single-agent ICIs. Over more than 4.5 years of follow-up, this disease has demonstrated remarkable sensitivity to single-agent ICIs. Most of our patients had germline Lynch syndrome, with a manageable safety profile. With more than 40% of patients remaining progression-free by the time of analysis, these observations appear favorable relative to historical outcomes in recurrent UTUC. Most patients with recurrent UTUC die within 3 years despite receiving chemotherapy, with 3-year CSS rates of 9.7% overall and 4.4% in patients with a poor performance status and liver metastasis.^8^

Existing evidence on response and outcomes of ICIs in UTUC is derived from small UTUC subsets within prospective trials and retrospective cohorts that report variable outcomes.^6^ In a large multi-institutional retrospective study, Esagian et al^9^ found that although overall outcomes with ICIs were comparable between advanced upper and lower tract UC (LTUC), mixed-histology UTUC had a significantly lower ORR and shorter PFS compared with mixed-histology LTUC. Our observed ORR of 83% also exceeds that reported in a phase 2 trial of pembrolizumab-based monotherapy for cisplatin-ineligible UC, in which only 26% of 69 patients with metastatic UTUC had responses.^10^

Our observed median treatment duration of 14.4 months (IQR: 4.7-25) reflects relatively prolonged exposure to single-agent ICIs. It is numerically longer than the typical treatment exposure reported in prospective pembrolizumab trials in metastatic UC, where many patients discontinue early due to progression or toxicity. In the KEYNOTE-052 phase II study of first-line pembrolizumab in cisplatin-ineligible LA or mUTUC, pembrolizumab was per *protocol* administered until progression or toxicity with a maximum of 24 months. However, around half of patients discontinued treatment early within the first 3 months and approximately 21% remained on treatment for at least 1 year.^11^ Similarly, in KEYNOTE-361, the median exposure in the pembrolizumab monotherapy arm was seven cycles (IQR: 3-17), with just over half of patients receiving at least six cycles.^12^ In the second-line setting, KEYNOTE-045 demonstrated an OS benefit for pembrolizumab over chemotherapy, although treatment duration was limited by disease progression in many patients.^13^ In LEAP-011, treatment duration was short and comparable in the lenvatinib plus pembrolizumab and the pembrolizumab-alone arm (approximately 4 months), and the addition of lenvatinib to pembrolizumab did not improve outcomes and was associated with higher toxicity, leading to increased treatment discontinuation.^14^ Collectively, these studies indicate that although early discontinuation is common, a subset of patients derive sustained benefit.^15^ The extended survival benefit in our cohort, including among patients who developed early irAEs, may reflect biologic enrichment within this specific molecular and anatomic subgroup.

Whereas EV plus pembrolizumab (EVP) is the current standard of care for LA or metastatic UTUC,^16^ our findings suggest that single-agent ICIs could serve as an alternative in certain patient subgroups, such as those with preexisting neuropathy. Additionally, with the long cancer-free durations observed, this approach might also offer the potential for kidney preservation to avoid dialysis or deferred surgery for surgically challenging cases.^3,17^ Given that 29.2% of patients experienced grade ≥3 irAEs leading to ICI discontinuation, mostly within the first 6 months, prolonged ICI therapy raises concerns about cumulative toxicity and financial and logistical burdens, highlighting the need to define optimal treatment duration.^18^

Although the EV-302 trial included 30.5% of patients with advanced UTUC, treatment with EVP significantly improved mPFS (12.3 *v* 6.2 months, hazard ratio [HR], 0.54 [95% CI, 0.38 to 0.76]) and mOS (36.5 months *v* 18.3 months, HR, 0.54 [95% CI, 0.37 to 0.78]) compared with chemotherapy in this subgroup. These benefits were consistent across other subpopulations, including lower tract disease, lymph node involvement, and presence or absence of hepatic metastases; however, the study was not designed to perform subgroup analyses based on MSI status.^19^ In a single-center retrospective series evaluating the radiographic response of intact primary lesions to EV or EVP in 20 response-evaluable patients with advanced UTUC, the ORR was 35% (7/20) and the disease control rate was 70% (14/20), with responses seen in both measurable and nontarget lesions, although MSI status was not assessed.^20^ Hence, the encouraging outcomes documented herein in the advanced setting, even among patients previously treated with systemic chemotherapy, warrant further investigation of the value of earlier introduction of single-agent ICIs in the resectable setting. This strategy is compelling given the higher pooled weighted prevalence of MSI-H in localized disease compared with metastatic disease (18.04% [95% CI, 13.36% to 23.91%] *v* 4.96% [95% CI, 2.72% to 8.86%], respectively).

Limitations of our study include its single-arm, nonrandomized design without a chemotherapy comparator, small sample size, retrospective single-center nature, and inherent susceptibility to selection and confounding biases. Data on tumor mutational burden (TMB) in our cohort were limited and warrant further exploration, especially given recent evidence suggesting improved survival with higher TMB in patients with advanced UC treated with ICIs in the maintenance setting.^21^ Standardized molecular testing of dMMR/MSI status in all patients with UTUC remains crucial with the advent of genomic sequencing.^22^ At our institution, all newly diagnosed patients with localized or advanced UTUC undergo tumor-based MSI and dMMR testing using both IHC and PCR. In cases with a suggestive family history, patients are referred to genetic counseling for further evaluation and germline testing. Somatic mutation testing is routinely performed on metastatic tumor biopsies in patients with advanced UC and is used here in an exploratory capacity to evaluate associations with putative germline alterations. However, immunohistochemical testing for dMMR remains essential in patients with localized disease due to its accessibility, cost-effectiveness, and ability to guide further genetic evaluation, including diagnosis of Lynch syndrome.

Germline pathogenic variants are identified in approximately 5%-24% of patients with UTUC, with Lynch syndrome MMR mutations accounting for about 5%-9%, alongside actionable mutations in other cancer predisposition genes such as ATM and BRCA1/2.^23,24^ Family history alone fails to capture nearly half of patients harboring hereditary mutations, underscoring the limitation of phenotype-based screening.^25^ In advanced UTUC, we advocate for universal tumor-based MMR testing, as MMR deficiency not only guides potential immunotherapy responsiveness but also carries important hereditary implications. Current NCCN Bladder Cancer Guidelines recommend that clinicians consider germline testing and referral to genetic counseling for patients with UTUC (ureteral or renal pelvis) who present at a younger age and/or have a personal or family history of Lynch syndrome–related cancers. Although our manuscript was not designed to evaluate the utility of germline testing for all patients with UTUC, any patient with an abnormal MMR/MSI result should be referred for genetic counseling and consideration of germline evaluation.

Finally, our findings generate the hypothesis that dMMR/MSI-H may represent a promising predictive and prognostic biomarker for UTUC and that first-line, single-agent ICIs like pembrolizumab could be beneficial for advanced disease in this context. Although dual ICI therapy with nivolumab plus ipilimumab has demonstrated superior efficacy to single-agent ICIs (nivolumab) in Lynch syndrome–associated CRC,^26^ there are currently no randomized trial data supporting dual checkpoint inhibition in urothelial carcinoma.^27^ Given the rarity of dMMR/MSI-H UTUC and the evolving treatment landscape in the era of ICI combinations, this observational study provides a benchmark for outcomes with single-agent ICIs in a well-defined molecular and anatomic subgroup. Future studies evaluating ICI-based combinations could contextualize outcomes in dMMR/MSI-H UTUC relative to this benchmark through biomarker-enriched cohorts or prespecified subgroup analyses. Prospective, multi-institutional studies are needed to validate these findings, evaluate the role of ICIs (single-agent or dual) in early-stage disease, and define strategies that maximize long-term efficacy while minimizing toxicity in dMMR/MSI-H UTUC.

As described in this report, we studied how well immunotherapy drugs like pembrolizumab worked when given as single agents to patients with advanced upper tract urothelial carcinoma, who had specific genetic signatures called deficient DNA mismatch repair and MSI, suspected to improve response to immunotherapy. Our findings demonstrated strong, long-lasting responses that appear to outperform the outcomes historically seen with traditional chemotherapy. We highlight the importance of testing for these genetic features in clinical practice, as it may guide therapy selection and help with referrals to genetic counseling.

## Data Availability

The datasets used and/or analyzed during the current study are available from the corresponding author on reasonable request.

## Notes

**Alexander Y. Andreev-Drakhlin**
**Employment:** Genentech/Roche **Stock and Other Ownership Interests:** Roche

**Aradhana Venkatesan**
**Other Relationship:** Dept. of Defense (Inst), Ingram Family Fund (UT MD Anderson Cancer Center) (Inst), Joint Center for Computational Oncology (UTMDACC Institute for Data Science in Oncology, UT Austin Oden Institute for Computational Engineering and Sciences & Texas Advanced Computing Center), MD Anderson Cancer Center (Inst)

**Ana C. Adriazola**
**Employment:** MD Anderson Cancer Center

## APPENDIX

**TABLE A1.** Definitions of Time-to-Event Outcomes in Our Analysis

Clinical Parameter	Definition and Details
Overall response rate	Proportion of patients with confirmed best overall response of CR or PR according to RECIST v1.1 criteria. Response was assessed based on at least one CT scan after initiation of single-agent ICI administration and confirmed upon subsequent imaging at least 6 weeks apart. Patients who died of complications of their cancer or were clinically determined to have disease progression before having imaging studies for response assessment were considered to have had PD
Clinical benefit rate	Proportion of patients having a best overall response of CR, PR, or SD lasting longer than 6 months
OS	Time from cycle 1 day 1 of therapy with ICIs to the date of death from any cause or date of last contact with a patient confirmed to be alive. OS was estimated using the Kaplan-Meier method (Prism, v10; GraphPad Software, Boston, MA, USA), and patients without an event were censored at their last documented follow-up visit
PFS	Time from start of therapy with ICIs to the date of disease progression or death from any cause. PFS was estimated using the Kaplan-Meier method. Intravesical recurrences or progression of NMIBC were not included
CSS	Time from initiation of therapy with ICIs to death attributed specifically to cancer. Patients who died from causes unrelated to cancer were censored at the time of death. CSS was estimated using the Kaplan-Meier method
Median follow-up time	Calculated for both OS and PFS using the reverse Kaplan-Meier method
12- and 24-months PFS rates (milestones)	Kaplan-Meier–estimated probabilities of remaining progression-free at 12 and 24 months after start of therapy with ICIs. Estimates were generated using R (version 4.3.2) with the survival package (version 3.5-7)
Time on treatment	Duration from start of therapy with ICIs to the date of the final administered dose
Duration of response	Time from the first documentation of an objective response (CR or PR) until the date of disease progression or death. The durations was estimated using the Kaplan-Meier method

Abbreviations: CT, computerized tomography; CR, complete response; CSS, cancer-specific survival; ICI, immune checkpoint inhibitor; NMIBC, nonmuscle invasive bladder cancer; PFS, progression-free survival; PR, partial response; SD, stable disease.

**TABLE A2.** Characteristics of Four Patients Who Underwent Surgical Consolidation After Treatment with ICIs

Pt No.	Mets Onset	DS	Primary Tumor	Pre-ICI CTx	BOR to CTx	BOR to ICI	ICI Tx Duration (no. Of Cycles)	Surgical Consolidation	Path Outcome	Reason of Surgical Consolidation
1	After initial diagnosis	LA/U	Left renal pelvis	No	—	SD	Pembrolizumab 5.4 months (10)	Left NU + RPLND	ypT1N0	Stage cT3 disease stabilized with ICI Tx
2	De novo	M	Left ureter	No	—	CR	Nivolumab 14.6 months (34)	Left NU + RPLND	mypT1N0	Noted enhancement along the renal pelvis and ureter, areas of prior disease after disease stability; definitive Tx selected
3	After initial diagnosis	LA/U	Left renal pelvis	ddMVAC	PD	PR	Pembrolizumab 2.2 months (4)	Left NU + RPLND	ypT0N0	LA disease stabilized with ICI Tx
4	After initial diagnosis	LA/U	Right renal pelvis	GTA then Gem/Cis	SD	CR	Pembrolizumab 4.4 months (6)	Right NU + RPLND	ypTaN0	Clinician's judgment of disease stability for bulky stage cT3 disease in the right collecting system + stable subcentimeter RP LNs

Abbreviations: BOR, best overall response; Cis, cisplatin; CR, complete response; CTx, chemotherapy; ddMVAC, dose-dense methotrexate, vinblastine, Doxorubicin, and cisplatin; DS, disease status; Gem, gemcitabine; GTA, gemcitabine, paclitaxel, and Doxorubicin; ICIs, immune checkpoint inhibitors; M, metastatic; Mets, metastasis; NED, no evidence of disease; NU, nephroureterectomy; Path, pathologic; Pt, patient; PR, partial response; RP LNs, retroperitoneal lymph nodes; SD, stable disease; Tx: treatment; U, unresectable.

**TABLE A3.** irAEs and Clinical Outcomes

irAE Event	irAE Leading to ICI d/c?	Time to irAE Onset from Tx Start	irAE Outcome	BOR to ICI
Pancytopenia (G3)	Yes	4.3 months	Consumptive immune destruction with cold agglutinin anemia: treated with corticosteroids and rituximab, resulting in recovery and cell count recovery	CR
Hepatitis and bullous pemphigoid (G4)	Yes	15.3 months	Resolution of hepatitis with mycophenolate mofetil, resolution of bullous pemphigoid with steroid taper and rituximab	PR
Colitis (G3)^a^	No	15.7 months	Resolved with FMT and vedolizumab; pembrolizumab successfully resumed thereafter	CR
Bullous pemphigoid (G3)	Yes	8.4 months	Maintained skin stability with dupilumab and topical betamethasone ointment	CR
Hepatitis (G3)	Yes	1.3 months	Resolved with a methylprednisolone dose pack	NE
Polyendocrinopathy (G3)	Yes	9.4 months	Persistent, requiring chronic corticosteroid replacement	CR
Bullous pemphigoid and nephritis (G3)	Yes	1.7 months	Bullous pemphigoid: original resolution then recurrent relapses managed with topical steroids; nephritis waxing and waning, stabilized with infliximab and preserved kidney function	SD
Dyspnea + polyarthritis + sarcoid-like reaction (G3)	Yes	3.3 months	Polyarthritis: stable with active surveillance; dyspnea: chronic with interval development of pulmonary fibrosis	SD
SICCA syndrome (G2)	Yes	19.67 months	Recurrent flares with dry lips and pruritus; each episode resolved with mouthwashes and short steroid tapers	CR

Abbreviations: BOR, best overall response; CR, complete response; d/c, discontinuation; FMT, fecal microbiota transplantation; G3/G4, grade 3/grade 4; ICI, immune checkpoint inhibitor; irAE, immune-related adverse event; NE, not evaluable; PR, partial response; SD, stable disease; tx, treatment.

^a^ With grade 2 pneumonitis.

**TABLE A4.** Reasons for Single-Agent ICI Treatment Discontinuation in Our Cohort

Reason for Treatment Discontinuation (n = 23)^a^	No. (%)
irAE	9 (37.5%)
Disease progression	1 (4.2%)
Surgical consolidation	4 (16.7%)
Completion per provider's choice or patient's preference	9 (37.5%)

Abbreviation: irAE, immune-related adverse event.

^a^ At the time of analysis, one patient (4.2%) remained on active treatment.
